# Efficacy of long-lasting insecticidal nets in use in Macha, Zambia, against the local *Anopheles arabiensis *population

**DOI:** 10.1186/1475-2875-10-254

**Published:** 2011-08-31

**Authors:** Laura C Norris, Douglas E Norris

**Affiliations:** 1 W. Harry Feinstone Department of Molecular Microbiology and Immunology, Johns Hopkins Bloomberg School of Public Health, 615 N. Wolfe St. Baltimore, MD 21218, USA

## Abstract

**Background:**

The mosquito *Anopheles arabiensis *is the primary vector of *Plasmodium falciparum *in Macha, Zambia. A major portion of Zambia's current malaria control programme relies on long-lasting insecticide-treated nets (LLINs) and indoor residual spraying (IRS) with insecticides. Currently, the efficacy of these measures against *An. arabiensis *in Macha is unknown, and previous data has shown that *An. arabiensis *has continued to feed on human hosts, despite high ITN coverage. It is possible that this could be due to either decreased efficacy of ITNs in used in Macha, or pyrethroid resistance in the vector.

**Methods:**

F1 offspring of field-collected adult *An. arabiensis *were tested for insecticide resistance, using CDC bottle bioassays and deltamethrin ITN susceptibility assays. The mosquitoes were characterized for the knock-down resistance (*kdr*) allele by PCR. LLINs that had been in use for two years in nearby villages were collected and tested for residual deltamethrin concentration and net quality, and were used in bioassays against susceptible colonized *Anopheles gambiae *s.s. Keele. Additionally, a survey on ITN use and care was conducted among LLIN owners.

**Results:**

In the F1 *An*. arabiensis field population, low levels of resistance to DDT and deltamethrin-treated net material were detected by bioassay, although the knock-down resistance (*kdr*) allele not present in the population. ITN evaluations revealed high variability in residual deltamethrin concentration, quality of the nets, and mosquito mortality in bioassays. Mortality against *An. gambiae *s.s. in bioassays was correlated with residual deltamethrin concentration, which was dependent upon the number of washes each net had received.

**Conclusions:**

Proper LLIN care was a strong determinant of LLIN efficacy, indicating that education on the importance of LLIN use and care is key when distributing nets. As there is little insecticide resistance in the local vector population, degradation of LLINs most likely allowed for continued human feeding by *An. arabiensis*. Continued monitoring and assessment of both the vector population and the efficacy of LLINs in use is necessary in order to appropriately modify vector control operations and prevent the development of pyrethroid resistance.

## Background

Malaria is a severe public health problem, causing an estimated 225 million disease cases and 781,000 deaths per year [1]. Most victims are children under five years old living in sub-Saharan Africa [1]. Malaria is transmitted by *Anopheles *mosquitoes, and because there is currently no vaccine available, vector control is one of the most important means of malaria prevention. Insecticide-treated nets (ITNs), introduced over 20 years ago [2,3], are an important tool to protect individuals against the morbidity and mortality caused by malaria [4,5]. ITNs can also decrease local malaria transmission by mass killing and decreased survival of anopheline vectors, thereby protecting those in the community without ITNs [6]. A more recent innovation is the long-lasting insecticide-treated net (LLIN), in which insecticide is either incorporated into the fiber during extrusion, or coated on the fiber or the finished net with a binding agent. Unlike conventional ITNs, which lose effective insecticide after one or two washes and last only 6-12 months, LLINs retain effective doses of insecticide up to 20 washes and have an expected lifespan of 3 to 4 years [7,8]. One LLIN recommended by the World Health Organization Pesticide Evaluation Scheme (WHOPES) is Permanet^®^, which contains 55 mg/m^2 ^deltamethrin ± 25% (minimum 45 mg/m^2^), and should retain 25 mg/m^2 ^deltamethrin after 6 standard washes, enough to still be effective against mosquitoes [9]. In laboratory tests, it provides 80% functional mortality for up to 20 laboratory washes [9].

Roll Back Malaria has set targets for global malaria control, including protecting 80% of at risk populations with ITNs or indoor residual spraying (IRS) by 2010 [10]. Zambia has had great success in rapidly scaling up malaria control measures [11,12]. In 2010, 73% of all households had at least one ITN or IRS within the last 12 months. Additionally, 52% of children under five years of age and 46% of pregnant women reported sleeping under an ITN the previous night [12]

Differences in use and wear in field settings may cause LLINs to lose residual insecticide more quickly, leading to loss of efficacy. A number of studies have examined the dynamics of insecticide loss and LLIN efficacy in field situations, with varying results. In Colombia, Permanet^® ^1.0 nets that had been washed 23 times over three years retained a mean of 9.6 mg/m^2 ^deltamethrin, and caused 79% mortality in *Anopheles *mosquitoes after three minutes of exposure in a WHO cone assay [13]. Similarly, a study in Kenya examined mean time to ITN failure, using < 50% mortality in WHO cone assays with *Anopheles gambiae *as a cutoff [14]. After two years of use, 82% of the Permanet^® ^1.0 LLINs were still effective, and the mean concentration of insecticide in failed nets was 11 mg/m^2^. In a multi-country trial of Permanet^® ^2.0 LLINs, the nets retained 13-24 mg/m^2 ^deltamethrin after 20 washes, and caused 80% mortality in *Anopheles stephensi *[15]. These studies all used standardized washing procedures. In contrast, a study in Ethiopia assayed Permanet^® ^2.0 LLINs used by villagers for two years without intervention. These nets maintained 67-72% mortality against *Anopheles arabiensis *[16]. A study of Permanet ^® ^2.0 LLINs in Uganda found 74% functional mortality after two years of household use in rural conditions [17].

In addition to insecticide loss, LLINs may lose efficacy if insecticide resistance develops in the mosquito target. Overuse of insecticides, whether in ITNs, indoor residual spraying, or in agricultural use, can select for resistance in mosquito vector species [18]. Resistance can be mediated either by mutations in the target site of the insecticide or its active metabolites (target-site resistance), or through enzymatic modification of insecticides to produce non-toxic metabolites (metabolic detoxification). DDT and pyrethroids, two types of insecticide approved for malaria control [19], share a common target, the para voltage-gated sodium channel. Knockdown resistance (*kdr*) mutations in the gene that codes for this channel can therefore confer cross-resistance to both DDT and pyrethroids that, when combined with metabolic resistance, can compromise insecticide-based malaria control efforts [20]. The *kdr *mutation has been shown to cause pyrethroid resistance in *An. gambiae *s.s. [21], and is present in several *An. arabiensis *populations in Africa: Kenya [22], South Africa [23], Burkina Faso [24], and Ethiopia [25].

Additionally, resistance to DDT and pyrethroids can be mediated through metabolic detoxification, by upregulation of cytochrome P450-dependent monooxygenases and glutathione S-transferases [18]. Phenotypic pyrethroid resistance in *An. arabiensis *has emerged in the Sudan [26], Mozambique [27], Uganda [28], and in Gwave, Zimbabwe [29], directly across the border from Southern Province, Zambia. DDT resistance in *An. arabiensis *has emerged in Sudan [26] and South Africa [23].

The Southern Province of Zambia has historically had hyperendemic *Plasmodium falciparum *malaria transmission [30] vectored primarily by *An. arabiensis*, with *An. funestus *as a secondary vector [31]. In the Choma District, where Macha is located, 61-80% of households have target coverage of 3 ITNs per household [11]. As part of the malaria control scale-up, a free mass distribution of ITNs by the Zambian government provided 4,800 LLINs to the Macha area in 2007, and 75% of individuals are protected by an ITN [32]. However, the pyrethroid susceptibility status of the *An. arabiensis *population in Southern Zambia is unknown.

There is 100% ITN coverage in households in Macha where mosquito surveillance collections are performed, yet 25-28% of *An. arabiensis *collected in CDC light traps are engorged, with human blood indices of 94-96% [32]. This indicates that despite high ITN use, *An. arabiensis *mosquitoes are still obtaining human blood meals. One explanation is mosquitoes feeding prior to 10 p.m., when residents go to bed, but only approximately 14% of *An. arabiensis *in Macha forage during this time [32]. An alternate explanation, explored in this study, is that the ITNs in use are not completely effective at preventing mosquito bites. This could be caused either by pyrethroid resistance in the *An. arabiensis *population, or by a loss of insecticide or holes in the LLINs. Therefore, our aim was to both investigate the insecticide susceptibility status of the *An. arabiensis *population in Macha, as well as to evaluate the condition of LLINs distributed in Macha, in order to determine their potential efficacy after two years of typical use in the community.

## Methods

### Study area

This study was conducted at the Johns Hopkins Malaria Research Institute's field station in Macha, Southern Province, Zambia. It is located at 16.39292°S, 26.79061°E, at an elevation of approximately 1,100 meters above sea level. The habitat around the Malaria Institute at Macha (MIAM) field station is characterized as Miombo woodland. Macha inhabitants are primarily subsistence farmers living in village areas under a headman. ITNs were collected from the villages of Chidakwa and Lupata, each 3 km from the research facility and Macha Mission Hospital. Each household in these villages consists of a collection of small houses constructed of mud or brick, with thatch or steel roofs. Previous surveys conducted during the rainy season have determined that approximately 75% of inhabitants in these villages slept under an ITN the previous night [32]. *Anopheles arabiensis *females were collected for insecticide resistance assays in Chidakwa, located 3 km from Macha hospital, and in Namwala, located 80 km north of Macha. Mosquitoes were collected during January 2010.

### *Anopheles arabiensis *colony

The MIAM *An. arabiensis *colony kept at Macha was characterized for insecticide susceptibility, and used as a baseline against which to compare field mosquitoes. This colony was established in February 2008 from mosquitoes collected in the Macha area. Because the colony has been through a strong bottleneck, and is kept free from any source of insecticide, it is presumed to be susceptible to most insecticides.

### F1 field mosquitoes

Anopheline mosquitoes were collected by outdoor human landing catch [33] and kept in paper cups with sugar solution. Mosquitoes were identified morphologically [34], and those identified as *An. gambiae *s.l. were kept in insectary conditions (28°C, 80% relative humidity) for blood feeding and oviposition. Mosquitoes were offered a blood meal from a mouse, then kept in individual snap-cap vials with 10% sucrose pads and damp filter paper for oviposition. Ethical approval for mouse feeds was given by the Johns Hopkins University Animal Care and Use Committee (Protocol Number: GP10H223). After oviposition, parental mosquitoes were killed by freezing, and DNA was extracted to identify species [35]. After parental mosquitoes were positively identified as *An. arabiensis*, F1 egg batches were pooled. Larvae were reared to adulthood in the insectary, and 3-5 day old mixed-sex adults were used in bioassays.

### CDC bottle bioassay

Adult mosquitoes were tested for insecticide susceptibility against permethrin, deltamethrin, DDT, and malathion (Sigma Aldrich, St. Louis, MO) using CDC bottle bioassays [36,37]. DDT was tested due to its use in indoor residual spraying programmes, permethrin and deltamethrin due to their use in LLINs and IRS, and malathion because it is used agriculturally. Briefly, the inside of 250-mL glass Wheaton bottles were coated with doses of insecticide diluted in acetone, including one control with acetone only. Dosages were measured as μg/bottle. The following dosages were tested against colony *An. arabiensis*: 30, 45, 60, and 75 μg/bottle permethrin; 50, 100, 150, and 200 μg/bottle DDT; 5, 10, 15, and 20 μg/bottle deltamethrin; 25, 50, 100, and 150 μg/bottle malathion. After the acetone had fully evaporated, 20-25 mosquitoes were introduced by aspiration. Knock-down was recorded at 15 minute intervals for three hours. After three hours, mosquitoes were removed from the bottles, separated into alive and knocked-down, and kept in separate paper cups with 10% sucrose solution for 24 hours in the insectary, after which they were scored as alive or dead. Diagnostic dosages established from these experiments were used to test the field population.

### Novel ITN susceptibility assay

Netting from a deltamethrin-treated Permanet^® ^LLIN was used to line the inside of a one-gallon cardboard container. A container lined with untreated netting material was used as a control for mortality. Twenty *An. gambiae *sensu strictu Keele laboratory colony mosquitoes were aspirated into each container and exposed to LLIN material for five minutes, after which they were gently shaken out into a cage and aspirated into labeled paper cups. They were then held for 24 hours in the insectary with 10% sucrose pads. After 24 hours, mosquitoes were categorized as dead or alive. Because pyrethroid insecticides cause mosquitoes to shed legs, which can negatively impact survival, live mosquitoes were further sorted into groups with 1-3 or 4-6 legs. This assay causes 100% mortality for deltamethrin-susceptible mosquitoes after 24 hours. The ITN assay was also compared against CDC bottle bioassays to determine mean time to knock down in both susceptible homozygous and *kdr *L1014F and L1014S mosquitoes (Rebecca Trout-Fryxell and Anton Cornell, unpublished data), and has been used to characterize deltamethrin-resistant *Culex quinquefasciatus *mosquitoes in Macha [38].

Like the WHO cone assay [39], this assay exposes mosquitoes to insecticide-treated netting material. However, in the WHO assay, mosquitoes are able to land on the cone and thus avoid the ITN material. This test is more conservative because all surfaces are covered in netting, forcing mosquitoes to land on the netting and become exposed to insecticide.

### Knockdown resistance (*kdr*) diagnostic

DNA was extracted from both colony and field samples of *An. arabiensis *using a modified salt-extraction [31] and was used to genotype samples for the *kdr *allele, using the PCR-based method of Tripet *et al *[40].

### LLIN collection

LLINs were collected in February 2009 from households that were known to have received Permanet^® ^2.0 LLINs from our field team during the ITN distribution in February 2007. Owners of the nets were surveyed about the nets and their use. Questions included: 1) How old is this net? 2) How many times have you washed the net? 3) How many/what size are the windows in the house where this net was hung? 4) What kind of fuel is burned in the house? 5) Was the net retreated? Households were provided with new Permanet^® ^3.0 nets to replace the nets taken. Nets were cataloged by their color and amount of wear, represented by the number of holes in five size categories (> 1 cm, 1-5 cm, 5-15 cm, 15-30 cm, > 30 cm). Nets were identified by the household number from which they were collected.

### Residual deltamethrin testing

20 cm × 20 cm samples were cut from the top, middle, and bottom of each net for residual deltamethrin quantitation by gas chromatography. Samples were weighed and placed in a 20 mL scintillation vial. 20 mL xylene and 150 μl internal standard solution (5 g/mL lambda-cyhalothrin in xylene) was added to each scintillation vial, which was capped and gently shaken. Vials were placed in a 90°C water bath for 2 hours, then sonicated for 15 minutes. After cooling, 50 μl of each sample solution was mixed with 950 μl xylene and transferred to an auto-injection vial. Sample solutions were injected into the gas chromatography system, alternating with a calibration solution of standard-grade deltamethrin.

### Field LLIN mosquito survival assays

Additional swatches were cut from the middle section of each bed net for mosquito survival bioassays. This assay was conducted similarly to the ITN susceptibility assay, using susceptible colonized *An. gambiae *s.s. Keele strain mosquitoes. Three trials were performed with each LLIN sample, for a total of 60 mosquitoes tested on each net.

### Statistics

The difference in deltamethrin concentration between top, middle, and bottom of bed net samples was tested by two-way ANOVA without replication. The effects of washing, wear on nets (measured by holes), type of fuel burned, and number of windows were analyzed as follows: washing data was categorized as no washes (0 reported washes), few washes (1-3 reported washes), and many washes (≥4 washes); the number of holes in each net was enumerated by size (< 1 cm, 1-5 cm, 5-15 cm, 15-30 cm, and > 30 cm) and the number of holes weighted by size was calculated to give a score for wear; nets were categorized as being in houses where either kerosene or an alternate fuel was burned (diesel, solar, flashlight, candles); and the number of windows was counted in each house, then categorized as few (1-3) or many (5-10). The concentration of deltamethrin from the middle of each net was log-transformed to give a normal distribution, and a multi-factorial ANOVA was performed with the above factors. Prior to all ANOVA analyses, data was tested for normality by the skewness/kurtosis test and for equal variance by Bartlett's test.

The relationship between deltamethrin concentration in middle swatches of the ITN and mosquito survival bioassays was modelled by logistic regression, after correcting for control mortality by Abbott's equation. The logistic regression was used to determine the LD_90 _of deltamethrin for this assay. All analyses were done in STATA 11 [41].

## Results

### CDC bottle bioassays

**Figure 1:**
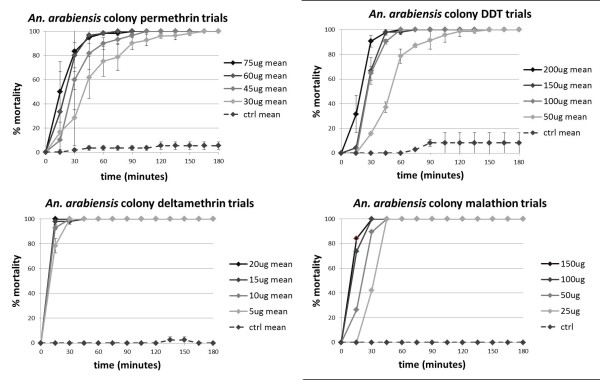
**Mortality curves for CDC bottle bioassays performed on the MIAM *An. arabiensis *colony**. In order to determine the diagnostic dosage, increasing concentrations of DDT, permethrin, deltamethrin, and malathion were used. The proper doses were determined to be: 100 μg/bottle DDT, 60 μg/bottle permethrin, 20 μg/bottle deltamethrin, and 100 μg/bottle malathion.

The diagnostic dosage for a CDC bottle bioassay is one that allows for discrimination between susceptible and resistant individuals. Dosages that are too low will show false resistance, while dosages that are too high will obscure actual resistance. Therefore, the diagnostic dosage is the lowest dose at which knock-down or mortality is saturated: increasing the dosage shows no increase in the slope of the mortality curve. Typically, this also correlates with 100% mortality within 1 hour. The following diagnostic dosages were established for the MIAM *An. arabiensis *colony: 60 μg/bottle permethrin, 100 μg/bottle DDT, 20 μg/bottle deltamethrin, and 100 μg/bottle malathion (Figure 1). These doses were close to the suggested CDC bottle bioassay doses for *Anopheles *species [42], supporting the hypothesis that this colony is susceptible to these insecticides. Different doses of deltamethrin, as low as 5 μg/bottle, had no effect on the slope of the mortality curve. Type I and II pyrethroids differ in their neurophysiological symptoms [43], and binding kinetics [20]. Deltamethrin, a Type II pyrethroid, is known to have high poisoning efficacy [44], so it is possible that its faster knockdown time made the effects of different doses indistinguishable in this assay. Therefore, 20 μg/bottle was used to test the field population, as this is a typical dose for *Anopheles *mosquitoes [42]. For all dosages tested, there was 100% mortality by 3 hours, and no recovery at the 24-hour time point.

**Figure 2:**
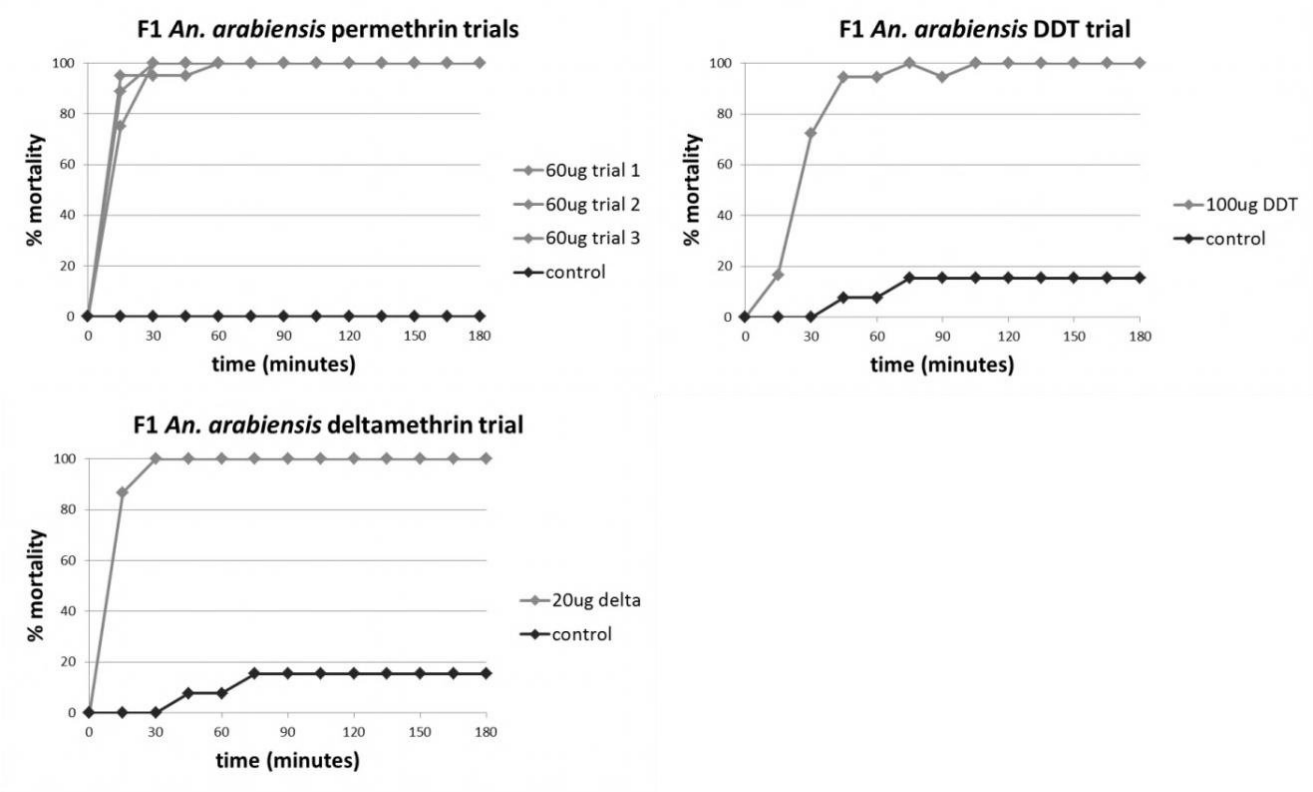
**Mortality curves for CDC bottle bioassays performed on F1 *An. arabiensis *from the field**. Doses of 60 μg/bottle permethrin, 20 μg/bottle deltamethrin, and 100 μg/bottle DDT were used. A small degree of DDT resistance, defined as < 100% mortality at 1 hour, was detected in the F1 *An. arabiensis *population.

The F1 field population was 100% susceptible to permethrin and deltamethrin at the diagnostic dosages used (Figure 2). In the DDT trial, mortality was 95% at 1 hour, and 100% mortality did not occur until 105 minutes (Figure 2). There was 100% mortality by 3 hours, and no recovery at the 24 hour time point. Due to a limited number of F1 mosquitoes and the priority of testing DDT, pyrethroids, and ITNs, F1 mosquitoes were not tested with malathion.

### ITN susceptibility assays

**Figure 3:**
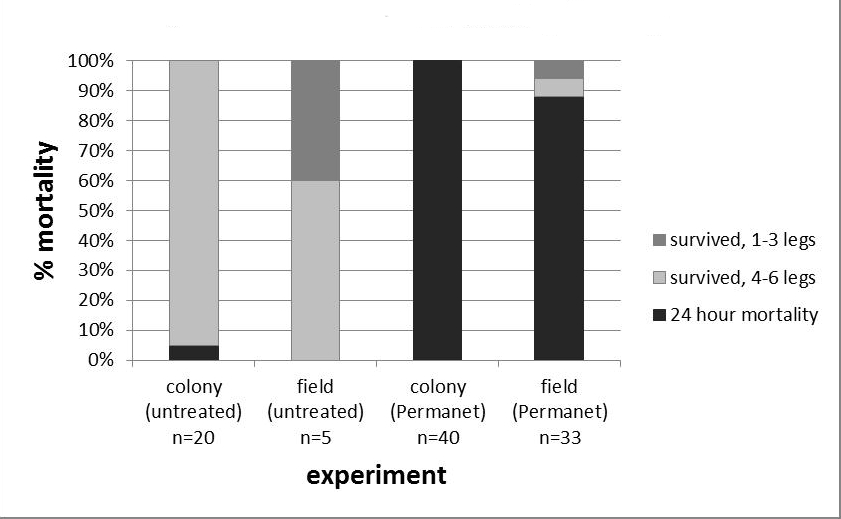
**ITN bioassay survival**. ITN survival assays were performed on MIAM *An. arabiensis *colony mosquitoes and F1 *An. arabiensis *from the field. Trials with untreated control netting and with deltamethrin-treated netting are shown. There was a small degree of resistance, defined as < 100% mortality at 24 hours, in the F1 field population.

At the 24 hour time point, colonized *An. arabiensis *showed 100% mortality when exposed to deltamethrin-treated Permanet^® ^material, versus 5% mortality with untreated material (Figure 3). In contrast, F1 field *An. arabiensis *showed only 88% mortality, with 6% surviving with 1-3 legs and 6% surviving with 4-6 legs. There was no mortality with untreated material, although 40% (2 out of 5) mosquitoes had 1-3 legs.

### *kdr *genotyping

In total, 50 colony *An. arabiensis*, 50 archived *An. arabiensis *from the field, and all 170 of the F1 *An. arabiensis *used in the bioassays were genotyped for *kdr*. All mosquitoes were homozygous wild-type, and the *kdr *L1014F and L1014S alleles were not present in the population.

### LLIN sampling

**Figure 4:**
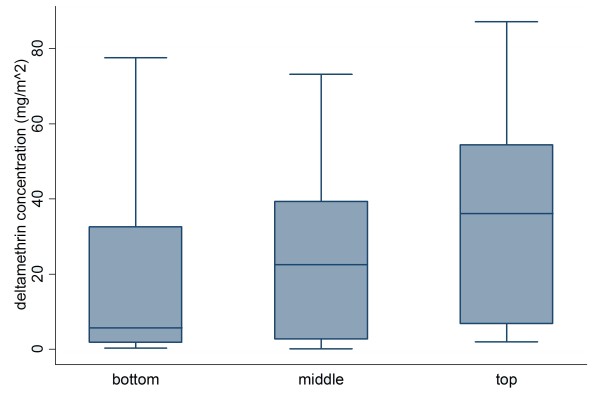
**Distribution of deltamethrin in sections from the top, middle, and bottom of LLINs**. Boxplots with median, range, quartiles, and outliers are shown. There were significantly lower residual deltamethrin concentrations in the middle and lower sections of the nets (p < 0.0001).

In total, 19 LLINs were collected from 16 households in Chidakwa and Lupata. All nets were currently hanging above beds in the houses when they were collected, indicating that they were actually in use. The median deltamethrin concentration varied depending on the location on the net from which the sample was taken, with 5.68 mg/m^2 ^for swatches from the bottom of nets, 22.5 mg/m^2 ^from the middle of nets, and 36.1 mg/m^2 ^from the top of nets (Figure 4). The difference between the top, middle, and bottom of nets was significant (p < 0.0001, two-way ANOVA). Deltamethrin concentrations from swatches from the middle of the nets were therefore taken representative of the whole net, in order to simplify further statistical analyses.

### Factors relating to deltamethrin retention

**Figure 5:**
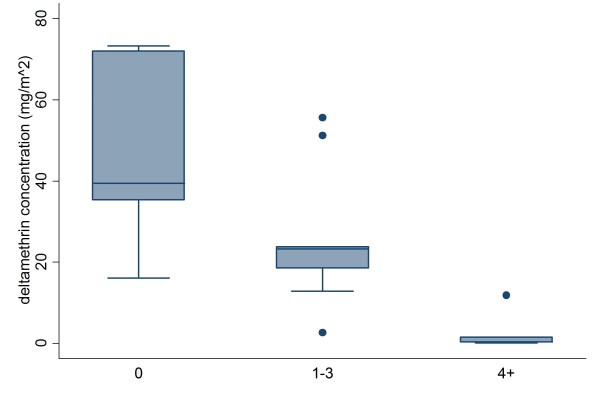
**Deltamethrin concentration in the middle sections of nets, sorted by 0, 1-3, and 4+ washes**. There was a significant correlation between number of washes and residual deltamethrin concentration (p = 0.001).

In the multi-factorial ANOVA, the only significant factor influencing deltamethrin concentration in bed nets was the number of reported washes (p = 0.001). Number of holes, which was a proxy for wear (p = 0.1608), kerosene burning in the hut (p = 0.9311) and number of windows (p = 0.9416) were not significant. There was wide variation in the reported number of times the LLINs were washed, with a minimum of 0 times over the 2 year period to a maximum of 24 times, with a median of two washes. The median deltamethrin concentration varied between wash categories, with 39.4 mg/m^2 ^for those with no washes, 23.3 mg/m^2 ^for those with 1-3 washes, and 0.38 mg/m^2 ^for those with 4 or more washes (Figure 5).

### Wear on LLINs

There was wide variability in the amount of wear on the LLINs (Table 1). Of the 19 nets, five had no holes of any size. The majority of holes were 1-5 cm in diameter, although five nets had holes greater than 15 cm in diameter, and one net had holes greater than 30 cm in diameter. Three of the nets had holes that had been mended by the owners.

**Table 1:** Number of holes in each LLIN, by diameter (centimeters)

Household ID	very small holes (< 1 cm)	small holes (1-5 cm)	medium holes (5-15 cm)	large holes (15-30 cm)	very large holes (> 30 cm)
**HH1-H2**			2	1	

**HH3-H5**		2		1	

**HH3-H8**		3			

**HH4-H5**		7	1		

**HH5-H1**			1		

**HH6-H2**		4	3		

**HH12-H3**					

**HH13-H2**					

**HH16-H2**					

**HH19-H7**		10	2		

**HH55-H8**		2	5	2	2

**HH58-H1**	2			1	

**HH70-H4**	15	10	1		

**HH75-H4**					

**HH75-H5**					

**HH79-H1**		9	2	4	

**HH81-H1**		1			

**HH90-H1**		1	1		

**HH90-H2**			1		

**Total**	**17**	**49**	**19**	**9**	**2**

### Field LLIN mosquito survival bioassays

**Figure 6:**
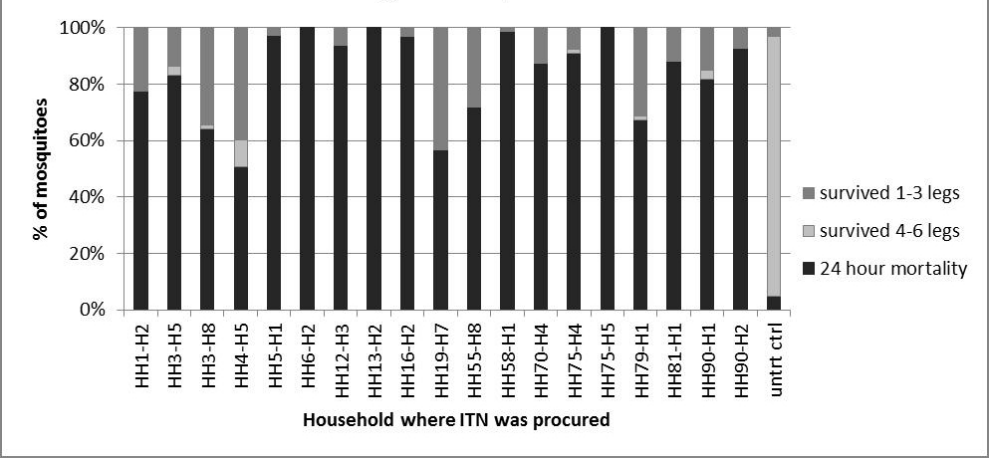
***An. gambiae *s.s. survival after exposure to netting obtained from LLINs in the field**. Outcomes are mortality after 24 hours, survival with 1-3 legs, or survival with 4-6 legs.

**Figure 7:**
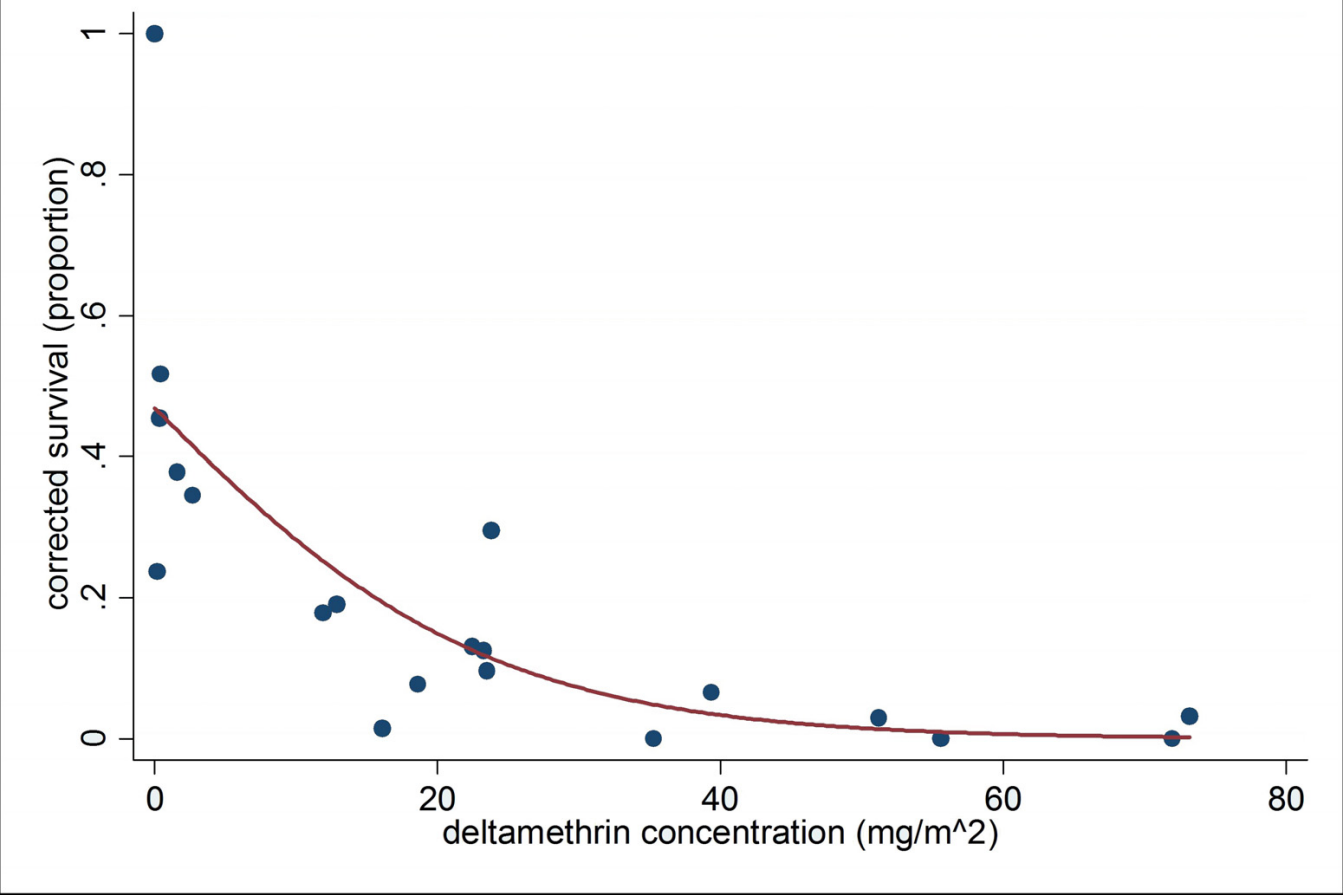
**Logistic regression: deltamethrin vs. mosquito survival in bioassays**. The relation between deltamethrin concentration in swatches of LLIN material and corrected mosquito survival in bioassays can be modelled as a logistic regression, using the function f(x) = 1/(1 + e^0.1253 + 0.0807x^) (p < 0.0001). The LD_90 _of deltamethrin-treated LLIN material is calculated as 25.7 mg/m^2^.

In the mosquito ITN survival bioassays, mortality ranged from 51% to 100%, with 4.8% mortality for control mosquitoes (Figure 6). The majority of surviving mosquitoes had 1-3 legs, although six nets had mosquitoes that survived with 4-6 legs, and for one net, 9.5% of all mosquitoes survived with 4-6 legs. For the negative control, 92% of all mosquitoes survived with 4-6 legs. The logistic model $f\left(x\right)=\frac{1}{1+e^{-\left(\beta_{0}+\beta_{1}x\right)}}$ was used to relate residual deltamethrin in the middle swatches of nets to mosquito survival in bioassays, with β_0 _= -0.1253 and β_1 _= -0.0807 (Figure 7). The model fit the observed data with p < 0.0001. Using this model, the LD_90 _for deltamethrin in this assay was calculated as 25.7 mg/m^2^.

## Discussion

The results of the CDC bottle bioassays with the MIAM *An. arabiensis *colony indicate that it is susceptible to permethrin, DDT, deltamethrin, and malathion, at dosages near those used to test *An. gambiae *s.s. It is also 100% susceptible to deltamethrin-treated material used in bed nets. Finally, the *kdr *allele, which causes resistance to pyrethroids and DDT, is absent from the colony. Therefore, the colony is appropriate to use as a susceptible control which can be used as a baseline against which to compare field populations. It can also be used for other applications, such as testing LLINs from the field for efficacy. This is the first report on this novel *An. arabiensis *colony, and the first such colony originating from this region.

The F1 field population of *An. arabiensis *appears to be completely susceptible to permethrin and deltamethrin, when tested in bottle bioassays. However, the mortality curve for the bottle bioassay with DDT shows slight resistance, and 12% of the mosquitoes tested survived after exposure to ITNs. It is possible that this test is more sensitive to lesser degrees of resistance in the mosquito. Because the *kdr *allele, which confers target-site resistance, is absent from the population, any resistance in this population is hypothesized to be mediated by metabolic detoxification. Metabolic detoxification is typically caused by gene amplification and transcriptional upregulation [18,45], making it more difficult to assay than *kdr*, which is a single nucleotide change. However, it can be assayed by detoxification enzyme activity assays [42]. Future studies will need to include characterization of the metabolic detoxification enzyme activity of this population of mosquitoes.

In contrast to previous research that focused on washing nets in a laboratory or tightly controlled field setting, this study examined how well LLINs performed in a real-world field setting, with no control or supervision over the behavior of the villagers using the bed nets. Therefore a wide range in variables were observed, such as the number of times a bed net was washed, how it was washed, UV exposure, handling and wear of the bed net, exposure to dust and soot, and other variables that may affect insecticide retention.

There was obvious patchiness in the distribution of residual deltamethrin in bed nets, with swatches from the bottom of the nets having the lowest concentration of insecticide. If ITNs are used improperly or not tucked under a mattress, mosquitoes and other biting insects can fly underneath the net, negating its effect as a physical barrier. Appropriate insecticide treatment, however, can either prevent this occurrence by spatial repellency, or cause the mosquito to die within 24 hours, preventing the spread of disease. If the bottom sections of nets do not retain enough residual insecticide, they may no longer have this protective effect. Similarly, holes and wear can cause nets to fail as a protective barrier, leaving only the effect of insecticide. If holes are very large, as was the case for some nets in this study, a mosquito can easily enter with very little searching, limiting the amount of insecticide contact, and again negating the ITN's protective effect.

ITNs, in addition to killing mosquitoes via insecticide, also function as a barrier to prevent bites. Holes in the net can undermine this function, particularly when residual deltamethrin concentrations in the net are too low to efficiently kill mosquitoes. It is therefore important to continue monitoring not only the concentration of insecticide in nets, but also their quality. A study in Kenya found that 40% of ITNs currently being used for malaria prevention were of poor quality due to the number of holes [46]. Although some of the nets in Macha had unacceptably large holes, a portion of them were completely intact after two years of use. The differences in wear between nets indicate that variation in how owners use nets can greatly affect bed net lifespan. It is possible that better education about how nets function to prevent malaria transmission by mosquitoes may help owners prioritize maintenance of their bed nets.

In this study, the greatest factor in loss of residual deltamethrin was the number of times the net was washed. It is possible that the other factors (wear, smoke, UV exposure) had an effect, but that it was not statistically significant due to the sample size, or that the proxies used to measure them did not accurately reflect the impact of those factors. Regardless, it is clear that the number of washes is not the only cause of deltamethrin loss. For example, some nets had never been washed, yet lost up to 30% of the residual insecticide from the top of the net, and up to 90% from the bottom of the net. It is possible that this variation was caused by the aforementioned factors, or by initial variation in the nets themselves. Nets that were washed at least once may have had additional variability in loss of deltamethrin, caused by differences in washing and drying methods between households.

The ITN survival bioassay used in this study is more conservative than the WHO cone test, because mosquitoes are forced to land on deltamethrin-treated netting, rather than having an untreated plastic surface available for resting. Despite this, some LLINs still had high survivorship when tested with the susceptible *An. gambiae s.s*. colony. For six of the nets, there was less than 80% mortality, and for six nets a proportion of mosquitoes survived with four to six legs. However, as with other characteristics, there were some nets that still retained high efficacy even after two years of use--three of the nets had 100% mortality against *An. gambiae *s.s. The logistic model showed that LLINs with deltamethrin concentrations as low as 25.7 mg/m^2 ^maintained a 90% mortality rate in the ITN susceptibility assays, which is extremely close to the threshold given by the manufacturer [9].

This study demonstrates the importance of education as a major component of vector control campaigns. Although the initial variation in insecticide concentration in the nets is unknown, Some of these bed nets retained sufficient insecticide after two years to be as efficacious as new, unused ITNs. Clearly, it is possible for these nets to perform in the field nearly as well as they perform in the lab. Educating communities about proper care of their LLINs (how frequently to wash them, how to properly use the net as a physical as well as chemical barrier, the importance of mending holes and keeping the net away from sunlight), could help provide them with truly long-lasting treated nets.

It is unclear whether the slight pyrethroid and DDT resistance in the *An. arabiensis *population will have an effect on vector control measures. If LLINs used in Macha are intact and have a full dose of residual deltamethrin, it is unlikely that this slight amount of resistance will have a great effect on LLIN efficacy. If, however, LLINs in Macha continue to deteriorate without replacement, insecticide resistance in the Macha *An. arabiensis *population may play a larger role. Bed nets with lower residual deltamethrin concentrations may allow for feeding and select for higher insecticide resistance. Ongoing surveillance in Southern Zambia, as well as other areas of Zambia, will be necessary to control pyrethroid resistance, if it does emerge. Additionally, bed nets with more and larger holes require less probing by mosquitoes for them to obtain a blood meal while avoiding a toxic dose of insecticide. It is likely that low deltamethrin concentration in LLINs and holes in the nets are allowing *An. arabiensis *mosquitoes to continue obtaining human blood meals in Macha despite the high use of ITNs.

## Conclusions

In Macha, there are currently no explicit plans for a mass redistribution of LLINs in the near future. Although ITN roll-out campaigns have had an admirable impact and provided protection to a vast number of people, it is important for the organizations involved to consider how best to sustain these campaigns in the future. Ongoing assessments of ITN efficacy in different locations are necessary to determine when ITNs should be replaced, and plans should be in place for how best to prepare for ITN failure. There have been several cases of countries with successful malaria control programmes where a dramatic resurgence of malaria cases was seen when control measures ceased, such a Sri Lanka [47], Zimbabwe [48], and Peru [49]. In particular, waning immunity due to decreased malaria rates can contribute to a resurgence after control fails. Only three years after the introduction of ITNs in Macha, ITN use has been correlated to decreased seropositivity for *P. falciparum *(Tamaki Kobayashi and William Moss, unpublished data). It is critical, therefore, that vector control measures be maintained to ensure the continuing success of malaria control in Macha.

## Competing interests

The authors declare that they have no competing interests.

## Authors' contributions

LN and DN conceived the study and designed the experiments. LN carried out mosquito and LLIN collections, mosquito bioassays, and PCRs, and analyzed the data. LN and DN drafted and wrote the manuscript. Both authors have read and approved the final manuscript.
